# Reducing the spread of artemisinin resistant malaria through community-level directly observed therapy in Western of Cambodia

**DOI:** 10.1186/1475-2875-11-S1-P86

**Published:** 2012-10-15

**Authors:** Chy Say, Sokomar Nguon, Soley Lek Dy, Kheang Soy Ty

**Affiliations:** 1 USAID Control and Prevention of Malaria Project, University Research Co. LLC, Phnom Penh, Cambodia; 2 National Malaria Control Program, Cambodia

## Background

Artemisinin resistant parasites have been documented recently in the Cambodia-Thailand border area, which has been the epicenter of parasite resistance to anti-malarials in the past. In September 2010, the USAID funded Malaria Control in Cambodia (MCC) project, in collaboration with National Malaria Control program (CNM) and with technical and financial support from WHO, conducted community Day-3 surveillance for Pf (+) to gather evidence of drug resistance in the area. From June, 2011, the community surveillance expanded follow up to Day-28.

## Methods

Twenty-six village malaria workers (VMWs) were trained on malaria screening and treatment through directly observed therapy (DOT). VMWs use RDT for all suspected malaria cases on Day-0, smears on Day-0 and Day-3, filled the case investigation form, and completed follow up on days 1-3, Day-7, and Day-28 if Day-3 (+). Both malaria smears are sent to the health center laboratory for reading on Day-3. Malaria patients are treated with DHA-PIP for three days with DOT. An SMS alert system is sent to a higher level if Day-3(+) is confirmed by lab staff. Cases identified positive on Day-7/Day-28 are referred for second line treatment with Quinine +Tetracycline for 7 days.

## Results

Between June 2011 and June 2012, 327 Pf positive cases were enrolled by VMWs. Over half (54%) were adult males between the ages of 15-49 years. A quarter (22%) were mobile and migrant workers. Twenty-seventy out of 327 cases (9%) remained positive on Day-3 and received further investigation and treatment. In addition, one case remained positive on Day-7, and 8 cases (30%) were positive on Day-28 as detailed in Table 1. The proportion of Day-3(+) cases decreased dramatically in the second phase of the pilot.

**Table 1:** Pf malaria cases enrolled in Tasanh health center catchment area on Day-0 to Day-28

Time frames	D0	D3 (%)	D7 (%)	D28 (%)
Sep 2010-May 2011	200	48 (24%)	n/a	n/a
Jun 2011-Jun 2012	327	27 (9%)	1 (4%)	8 (30%)
Total	527	75 (15%)		

Early warning signs of drug resistance detected by VMWs should be treated with second line treatment at the health facility, but only three referred cases were accepted for 2^nd^ line treatment. A challenge to seven-day treatment with Quinine + Tetracycline is the accessibility of health facilities. The CNM decided to implement other interventions such as IRS, FSAT, and screening surrounded index cases but they also have challenges.

## Conclusion and discussions

This pilot proves that malaria community DOT and follow up is vital to reduce the spread of potential multi-drug resistance. Identification of early warning signs of drug resistance and treatment by DOT for first line and second line by VMWs is a feasible approach. To ensure high quality results, strong monitoring, supervision, real-time feedback results are needed. Further discussion is needed to: (1) develop a comprehensive strategy to expand surveillance on a large scale including improving malaria case management and follow-up, (2) develop harmonized guidelines for promoting cross-border treatment and contact tracing, and (3) simplify the strategy for second line treatment and screening surrounding an index case.

